# Access to healthcare services for people recovering from alcohol excess

**DOI:** 10.1186/1472-6955-14-S1-S6

**Published:** 2015-10-08

**Authors:** Sarah Rhynas

**Affiliations:** 1 School of Health in Social Science, University of Edinburgh, EH8 9JT, UK

## Background

People recovering from alcohol excess can struggle to access healthcare services. Understandings of recovery need to be clarified in order to ensure services meet the needs of socially marginalised service users. In Scotland, alcohol excess is estimated to cost £3.6bn annually (Scottish Government 2015) [1]. Ensuring that service users can access appropriate services is a national priority.

## Aims

To explore the lives and give voice to people marginalised by alcohol related harm. To understand their experiences, support needs and relationships with the community.

## Methods

The qualitative participatory approach of Photovoice (Wang & Burris 1997) [2] was used to allow participants to use photography to tell their stories. The photos guided subsequent focus groups. Data were thematically analysed with input from participants. Findings were shared with policy-makers through exhibitions, events, conferences and writing.

## Results

Participants shared insights into the importance of belonging to the community, contributing and negotiating the challenges of life in recovery. The value of peer support in preference to traditional health and social care services was emphasised. The importance of designing responsive services which improve the accessibility of healthcare for marginalised groups was highlighted.

## Conclusions

Understanding the lives of people in recovery helps shape services. Providing peer support services within therapeutic social environments may be preferred by service users, ensuring their needs are met and healthcare is more accessible. Alcohol addiction is a national priority in Scotland but is relevant throughout Europe. Ensuring that services are accessible to users requires detailed understanding of their lives.
